# Potential use of *Helianthus tuberosus* to suppress the invasive alien plant *Ageratina adenophora* under different shade levels

**DOI:** 10.1186/s12862-021-01826-5

**Published:** 2021-05-16

**Authors:** Shicai Shen, Gaofeng Xu, Diyu Li, Shaosong Yang, Guimei Jin, Shufang Liu, David Roy Clements, Aidong Chen, Jia Rao, Lila Wen, Qiong Tao, Shuiying Zhang, Jiazhen Yang, Fudou Zhang

**Affiliations:** 1 Key Laboratory of Green Prevention and Control of Agricultural Transboundary Pests of Yunnan Province, Agricultural Environment and Resource Research Institute, Yunnan Academy of Agricultural Sciences, Kunming, 650205 China; 2 Biology Department, Trinity Western University, 7600 Glover Road, Langley, BC V2Y 1Y1 Canada; 3 Agricultural Biotechnology Key Laboratory of Yunnan Province, Biotechnology and Germplasm Resources Institute, Yunnan Academy of Agricultural Sciences, Kunming, 650205 China

**Keywords:** *Helianthus tuberosus*, *Ageratina adenophora*, Shade levels, Competitive interactions, Growth suppression, Net photosynthetic rate

## Abstract

**Background:**

An ecological approach for managing biological invasions in agroecosystems is the selection of alternative crop species to manage the infestation of invasive alien plants through competition. In the current study, plant growth, photosynthesis, and competitive ability of the crop *Helianthus tuberosus* L. (Jerusalem artichoke) and the invasive alien plant *Ageratina adenophora* (Spreng.) R. M. King and H. Rob were compared under varying shade levels by utilizing a de Wit replacement series method. We hypothesized that *H. tuberosus* had higher competitive ability than *A. adenophora* even under shaded conditions.

**Results:**

The results showed the main stem, leafstalk length, leaf area, underground biomass, and aboveground biomass of *A. adenophora* were significantly lower compared to *H. tuberosus* in monoculture although *A. adenophora* had a greater number of branches that were longer on average. Under full sunlight, the total shoot length (stem + branch length), main stem length and branch length of *A. adenophora* were significantly suppressed (P < 0.05) by increasing proportions of *H. tuberosus*, and the same morphological variables of *H. tuberosus* were significantly higher with decreasing proportions of *H. tuberosus.* With increasing shade rates and plant ratios, the plant height, branch, leaf, and biomass of both plants were significantly suppressed, but to a greater degree in the case of *A. adenophora.* The net photosynthetic rate (Pn) of *H. tuberosus* and *A. adenophora* increased gradually from July to September, then decreased in October. The Pn of *H. tuberosus* was consistently higher than that of *A. adenophora.* Although the Pn for both species was significantly reduced with increasing shade rates and plant ratios, *A. adenophora* experienced greater inhibition than *H. tuberosus*. The relative yield (RY) of *A. adenophora* was significantly less than 1.0 (P < 0.05) in mixed culture under all shade levels, indicating that the intraspecific competition was less than interspecific competition. The RY of *H. tuberosus* was significantly less than 1.0 under 40*–*60% shade and greater than 1.0 (P < 0.05) under 0*–*20% shade in mixed culture, respectively, showing that intraspecific competition was higher than interspecific competition under low shade, but the converse was true under high shade. The relative yield total (RYT) of *A. adenophora* and *H. tuberosus* was less than 1.0 in mixed culture, indicating that there was competition between the two plants. The fact that the competitive balance (CB) index of *H. tuberosus* was greater than zero demonstrated a higher competitive ability than *A. adenophora* even at the highest shade level (60%).

**Conclusions:**

Our results suggest that *H. tuberosus* is a promising replacement control candidate for managing infestations of *A. adenophora*, and could be widely used in various habitats where *A. adenophora* invades.

## Background

Jerusalem artichoke (*Helianthus tuberosus* L.), also known as sunchoke, is a perennial herbaceous plant from family Asteraceae [1]. Native to North America, this plant has become broadly distributed throughout the world and introduced into China via Europe [2]. As an important multifunctional crop, *H. tuberosus* has been widely utilized in agriculture and industry. This crop usually produces around 7 t and potentially up to 14 t ha^−1^ of carbohydrate [3]. Its aerial parts and tubers are used as high quality fodder for livestock [4]. Tubers of *H. tuberosus* contain abundant inulin, B vitamins, pantothenic acid, potassium, phosphorus, vitamin A, iron, and calcium, providing an excellent vegetable and food source for human diets [5, 6]. The crop is used for the production of paper pulp, fuelwood, methane acetone, butanol, ethanol, hydroxymethylfurfural, fodder yeast, beer, lactic acid, propionic acid, mannitol, and pectic substances in industry [1, 3, 4, 6, 7]. Moreover, it may be grown to stabilize unstable sand and terraces, to provide fire barriers in forests, or as a promising crop for planting in coastal marginal land in China [6, 8–10].

*Helianthus tuberosus* has strong tolerance and suitablilty to various environmental and climatic conditions allowing it to be easily grown in tropical, temperate, frigid, and even arid and semi-arid regions [8–10]. Recently this plant has been emerging as an important economic crop in China. *Helianthus tuberosus* is known to have a strong competitive advantage through its efficient use of sunlight, stress resistance and vegetative propagation ability in comparison to other plants [4]. This crop has been demonstrated to suppress growth and photosynthetic ability of several invasive plants such as *Ambrosia trifida*, *Cenchrus pauciflorus*, and *Flaveria bidentis* [11–13]. Furthermore, allelochemicals produced by *H. tuberosus* may interfere with the growth of other species, resulting in improved growth, development, and spread by *H. tuberosus* [14, 15]. Therefore, this crop exhibits great potential to provide ecological management of other invasive alien plants.

Previous observations in fields where *H. tuberosus* was grown in Yunnan Province, China, indicated that *H. tuberosus* appeared to compete strongly with the invasive alien plant *Ageratina adenophora* (Spreng.) R. M. King and H. Rob. Native to Mexico and Costa Rica in Central America, *A. adenophora* has been considered one of the most problematic invasive alien species globally [16, 17]. In China, this invasive plant was first introduced from Myanmar into the south Lincang of Yunnan Province in the 1940s, and is now widely distributed in southwest regions of the country [18, 19]. *Ageratina adenophora* has invaded a broad range of habitats, causing tremendous economic losses and negative environmental and biodiversity impacts [20, 21]. This weed is a heliophilic species, but it may still grow under low sunlight conditions [22]. *Ageratina adenophora* retained advantages over two native congeners across different light levels, showing its greatest advantage under light saturated conditions, with its relative performance decreasing at lower irradiance levels [23]. Moreover, plasticity in some of these physiological traits may play a role in invasion success for *A. adenophora* but varies in different environments making broad generalizations difficult [24]. Thus, there is an urgent need to explore more effective control methods for mitigating the damage caused by the invasion of *A. adenophora*, including crop plants that could reduce *A. adenophora* populations through competition even under shaded conditions.

Based on preliminary field observations of excellent inhibition of *A. adenophora* by *H. tuberosus*, the main objective of this study was to examine the competitive relationship between *H. tuberosus* and *A. adenophora*, by looking at plant growth and photosynthesis characteristics under different shade levels in order to provide a scientific basis for setting up an effective management method utilizing ecological control techniques for *A. adenophora*.

## Results

### Plant growth

Plant growth of *H. tuberosus* and *A. adenophora* was significantly affected (P < 0.01) by the shade rates and density ratios (Tables 1, 2). In general, the main stem length of *H. tuberosus* was markedly longer than that of *A. adenophora,* but its branch length was less than that of *A. adenophora*. Under full sunlight conditions, the total shoot length (stem + branch length), main stem length and branch length of *A. adenophora* were significantly suppressed (P < 0.05) with increasing proportions of *H. tuberosus*. The main stem length and branch length of *A. adenophora* were reduced by 51.5% and 76.2% at the 2:1 *H. tuberosus*: *A. adenophora* ratio in mixed culture (Table 1). The main stem length (except 20% shade for *A. adenophora*) and branch length of *H. tuberosus* and *A. adenophora* were significantly suppressed with increasing shade rates in mono and mixed culture with *A. adenophora* generally more inhibited (Table 2). During the experiment, following the initial sprouting of *H. tuberosus* about one week after transplantation, its growth rate accelerated. Plant height of *H. tuberosus* exceeded that of *A. adenophora* within two weeks. The percent cover of *H. tuberosus* reached 75% at 50 days, and exceeded 95% within 60*–*65 days. By comparison, the percent cover of *A. adenophora* was only about 40% at 65 days, even in monoculture.

**Table 1:** Morphological characteristics and biomass of *Helianthus tuberosus* and *Ageratina adenophora* competition under full sunlight

Variables	Ratios (*H. tuberosus*: *A. adenophora*)				
	4:0	2:1	1:1	1:2	0:4
Total shoot length (cm)					
* H. tuberosus*	173.62 ± 3.59c	181.74 ± 4.37b	190.54 ± 4.51ab	197.62 ± 4.26a	–
* A. adenophora*	–	61.67 ± 1.77d	96.11 ± 1.46c	140.58 ± 1.80b	171.39 ± 2.26a
Main stem length (cm)					
* H. tuberosus*	138.06 ± 2.68c	143.17 ± 3.76bc	149.34 ± 3.60ab	152.96 ± 3.27a	–
* A. adenophora*	–	41.06 ± 1.68d	60.79 ± 1.55c	75.22 ± 1.25b	84.72 ± 1.93a
Total branch length (cm)					
* H. tuberosus*	35.56 ± 1.13d	38.58 ± 1.09c	41.19 ± 1.09b	44.66 ± 1.04a	–
* A. adenophora*	–	20.61 ± 0.47d	35.42 ± 0.50c	65.36 ± 0.65b	86.67 ± 0.96a
Branch number					
* H. tuberosus*	3.5 ± 0.4b	4.0 ± 0.4b	4.9 ± 0.5a	5.1 ± 0.5a	–
* A. adenophora*	–	6.4 ± 0.5d	9.4 ± 0.5c	14.3 ± 0.6b	18.3 ± 0.9a
Leafstalk length (cm)					
* H. tuberosus*	5.80 ± 0.02c	5.95 ± 0.04b	6.06 ± 0.03a	6.10 ± 0.03a	–
* A. adenophora*	–	3.01 ± 0.02d	3.45 ± 0.01c	3.83 ± 0.01b	4.74 ± 0.02a
Leaf area (cm^2^)					
* H. tuberosus*	64.57 ± 0.59c	65.21 ± 0.71bc	66.18 ± 0.74b	67.93 ± 0.70a	–
* A. adenophora*	–	16.20 ± 0.15d	20.25 ± 0.16c	33.32 ± 0.36b	38.34 ± 0.24a
Root biomass (g)					
* H. tuberosus*	236.97 ± 2.94c	262.79 ± 4.60b	264.76 ± 3.38b	286.44 ± 3.20a	–
* A. adenophora*	–	2.74 ± 0.09d	4.20 ± 0.24c	8.25 ± 0.27b	14.32 ± 0.30a
Aboveground biomass (g)					
* H. tuberosus*	95.16 ± 1.00c	105.86 ± 2.03b	106.75 ± 3.43b	125.40 ± 3.98a	–
* A. adenophora*	–	11.24 ± 0.29d	14.31 ± 0.29c	16.29 ± 0.36b	42.34 ± 1.43a
Total biomass (g)					
* H. tuberosus*	332.13 ± 3.79c	368.64 ± 4.94b	371.50 ± 5.94b	411.84 ± 5.77a	–
* A. adenophora*	–	13.98 ± 0.20d	18.51 ± 0.24c	24.54 ± 0.41b	56.64 ± 1.21a

Data are expressed as mean ± standard deviation. Different letters within the same row signify significant differences at P < 0.05

**Table 2:** Morphological characteristics and biomass of *Helianthus tuberosus* and *Ageratina adenophora* competition under different shade levels

**Variables**	**Different shade rates**			
	**60%**	**40%**	**20%**	**0%**
Total shoot length (cm)				
* H. tuberosus*				
* H. tuberosus*: *A. adenophora* = 4:0	95.51 ± 1.43d	116.10 ± 2.44c	136.04 ± 2.16b	173.62 ± 3.59a
* H. tuberosus*: *A. adenophora* = 1:1	93.71 ± 1.85d	119.56 ± 1.47c	158.94 ± 3.22b	190.54 ± 4.51a
* A. adenophora*				
* H. tuberosus*: *A. adenophora* = 1:1	45.67 ± 0.53d	53.20 ± 0.60c	72.33 ± 1.40b	96.21 ± 1.46a
* H. tuberosus*: *A. adenophora* = 0:4	73.26 ± 1.65d	98.03 ± 1.55c	164.42 ± 3.06b	171.39 ± 2.26a
Main stem length (cm)				
* H. tuberosus*				
* H. tuberosus*: *A. adenophora* = 4:0	84.27 ± 1.66d	99.23 ± 2.32c	108.31 ± 2.04b	138.06 ± 2.68a
* H. tuberosus*: *A. adenophora* = 1:1	79.98 ± 1.75d	94.11 ± 1.41c	126.48 ± 3.35b	149.34 ± 3.60a
* A. adenophora*				
* H. tuberosus*: *A. adenophora* = 1:1	32.52 ± 0.57d	37.96 ± 0.64c	50.28 ± 0.97b	60.79 ± 1.55a
* H. tuberosus*: *A. adenophora* = 0:4	56.78 ± 1.42d	67.31 ± 1.13c	111.06 ± 2.08a	84.72 ± 1.93b
Total branch length (cm)				
* H. tuberosus*				
* H. tuberosus*: *A. adenophora* = 4:0	11.24 ± 0.31d	16.87 ± 0.19c	27.73 ± 0.33b	35.56 ± 1.13a
* H. tuberosus*: *A. adenophora* = 1:1	13.72 ± 0.18d	25.45 ± 0.34c	32.46 ± 0.36b	41.19 ± 1.09a
* A. adenophora*				
* H. tuberosus*: *A. adenophora* = 1:1	13.16 ± 0.46d	15.24 ± 0.25c	22.06 ± 0.60b	35.42 ± 0.50a
* H. tuberosus*: *A. adenophora* = 0:4	16.49 ± 0.27d	30.72 ± 0.48c	53.37 ± 1.00b	86.67 ± 0.96a
Branch number				
* H. tuberosus*				
* H. tuberosus*: *A. adenophora* = 4:0	1.9 ± 0.3b	2.1 ± 0.3b	2.8 ± 0.6a	3.5 ± 0.4a
* H. tuberosus*: *A. adenophora* = 1:1	2.5 ± 0.4b	2.8 ± 0.3b	3.4 ± 0.5ab	4.9 ± 0.5a
* A. adenophora*				
* H. tuberosus*: *A. adenophora* = 1:1	3.4 ± 0.3d	4.2 ± 0.5c	6.1 ± 0.3b	9.4 ± 0.5a
* H. tuberosus*: *A. adenophora* = 0:4	4.5 ± 0.4d	7.4 ± 0.5c	13.4 ± 0.5b	18.3 ± 0.9a
Leafstalk length (cm)				
* H. tuberosus*				
* H. tuberosus*: *A. adenophora* = 4:0	5.36 ± 0.02c	5.50 ± 0.04b	5.78 ± 0.02a	5.80 ± 0.02a
* H. tuberosus*: *A. adenophora* = 1:1	5.04 ± 0.03d	5.37 ± 0.01c	5.82 ± 0.02b	6.06 ± 0.03a
* A. adenophora*				
* H. tuberosus*: *A. adenophora* = 1:1	3.19 ± 0.02d	3.31 ± 0.02c	3.37 ± 0.01b	3.45 ± 0.01a
* H. tuberosus*: *A. adenophora* = 0:4	3.94 ± 0.02d	4.04 ± 0.02c	4.47 ± 0.01b	4.74 ± 0.02a
Leaf area (cm^2^)				
* H. tuberosus*				
* H. tuberosus*: *A. adenophora* = 4:0	52.26 ± 0.13d	55.11 ± 0.12c	60.11 ± 0.17b	64.57 ± 0.59a
* H. tuberosus*: *A. adenophora* = 1:1	51.88 ± 0.13d	56.36 ± 0.21c	61.75 ± 0.19b	66.18 ± 0.74a
* A. adenophora*				
* H. tuberosus*: *A. adenophora* = 1:1	16.21 ± 0.21d	17.13 ± 0.15c	19.19 ± 0.13b	20.25 ± 0.16a
* H. tuberosus*: *A. adenophora* = 0:4	26.23 ± 0.20d	30.28 ± 0.14c	35.22 ± 0.20b	38.34 ± 0.24a
Root biomass (g)				
* H. tuberosus*				
* H. tuberosus*: *A. adenophora* = 4:0	95.31 ± 2.04d	155.03 ± 2.75c	200.62 ± 3.68b	236.97 ± 2.94a
* H. tuberosus*: *A. adenophora* = 1:1	95.51 ± 1.89d	144.01 ± 1.66c	205.97 ± 2.50b	264.76 ± 3.38a
* A. adenophora*				
* H. tuberosus*: *A. adenophora* = 1:1	1.38 ± 0.03d	2.25 ± 0.09c	2.75 ± 0.09b	4.20 ± 0.24a
* H. tuberosus*: *A. adenophora* = 0:4	3.22 ± 0.16d	4.67 ± 0.21c	9.68 ± 0.49b	14.32 ± 0.30a
Aboveground biomass (g)				
* H. tuberosus*				
* H. tuberosus*: *A. adenophora* = 4:0	67.78 ± 1.46d	72.30 ± 1.77c	90.90 ± 1.26b	95.16 ± 1.00a
* H. tuberosus*: *A. adenophora* = 1:1	64.61 ± 1.09d	73.97 ± 1.03c	91.13 ± 1.89b	106.75 ± 3.43a
* A. adenophora*				
* H. tuberosus*: *A. adenophora* = 1:1	3.33 ± 0.26d	5.55 ± 0.36c	8.96 ± 0.35b	14.31 ± 0.29a
* H. tuberosus*: *A. adenophora* = 0:4	7.30 ± 0.16d	13.63 ± 0.49c	21.94 ± 0.62b	42.34 ± 1.43a
Total biomass (g)				
* H. tuberosus*				
* H. tuberosus*: *A. adenophora* = 4:0	163.08 ± 2.86d	227.33 ± 2.58c	291.52 ± 4.52b	332.13 ± 3.79a
* H. tuberosus*: *A. adenophora* = 1:1	160.12 ± 1.43d	217.98 ± 2.49c	297.10 ± 1.68b	371.50 ± 5.94a
* A. adenophora*				
* H. tuberosus*: *A. adenophora* = 1:1	4.71 ± 0.25d	7.79 ± 0.33c	11.72 ± 0.44b	18.51 ± 0.24a
* H. tuberosus*: *A. adenophora* = 0:4	10.53 ± 0.17d	18.29 ± 0.41c	31.61 ± 0.84b	56.64 ± 1.21a

Data are expressed as mean ± standard deviation. Different letters within the same row signify significant differences at P < 0.05

The branch number of *A. adenophora* was much higher than that of *H. tuberosus* in monoculture (Tables 1, 2)*.* Under full sunlight conditions, the branch number of *A. adenophora* was significantly suppressed (P < 0.05) with decreasing proportions of *A. adenophora*, and that of *H. tuberosus* was increased markedly with increasing proportions of *A. adenophora* in mixed culture; and the branch number of *A. adenophora* was inhibited by 65.0% at the 2:1 *H. tuberosus*: *A. adenophora* ratio in mixed culture (Table 1). The branch number of *H. tuberosus* and *A. adenophora* was significantly reduced with increasing shade rates, and the inhibition rates of *A. adenophora* were significantly higher (P < 0.05) than those of *H. tuberosus* in mono and mixed culture (Tables 1, 2).

The leafstalk length and leaf area of *H. tuberosus* were markedly greater than those of *A. adenophora* in all treatments (Tables 1, 2). Under full sunlight conditions, the mean leafstalk length and leaf area of *H. tuberosus* were 5.80 cm and 64.57 cm^2^, respectively, whereas those of *A. adenophora* were only 4.74 cm and 38.34 cm^2^, respectively in monoculture. The leafstalk length and leaf area of *A. adenophora* progressively declined (P < 0.05) with increasing proportions of *H. tuberosus*, and those of *H. tuberosus* were significantly increased with increasing proportions of *A. adenophora* in mixed culture. The leafstalk length and leaf area of *A. adenophora* were reduced by 36.5% and 57.7% at the 2:1 *H. tuberosus*: *A. adenophora* ratio in mixed culture, respectively (Table 1)*.* The leafstalk length and leaf area of *H. tuberosus* and *A. adenophora* were significantly reduced with increasing shade rates in mono and mixed culture, and both shade and plant competition inhibited these parameters more for *A. adenophora* (Table 2).

The total biomass of *H. tuberosus* was much greater than that of *A. adenophora* in all treatments (Table 1). Under full sunlight conditions, the underground biomass and aboveground biomass of *A. adenophora* were significantly suppressed (P < 0.05) with decreasing proportions of *A. adenophora*, whereas the biomass of *H. tuberosus* was markedly increased with increasing proportions of *A. adenophora* in mixed culture. The underground biomass and aboveground biomass of *A. adenophora* were reduced by 80.9% and 73.5% at the 2:1 *H. tuberosus*: *A. adenophora* ratio in mixed culture, respectively (Table 1)*.* Under shaded conditions, the underground biomass and aboveground biomass of *H. tuberosus* and *A. adenophora* were significantly reduced with increasing shade rates, inhibiting *A. adenophora* were higher than those of more than *H. tuberosus* in mono and mixed culture (Table 2).

### Photosynthesis

The photosynthetic rate (Pn) of both *H. tuberosus* and *A. adenophora* increased gradually from July to September, then decreased in October in all treatments. The Pn of *H. tuberosus* from July to October was higher than that of *A. adenophora* (Tables 3, 4). Under shaded conditions, the Pn of *H. tuberosus* was significantly higher than that of *A. adenophora*, and there were few differences within treatments for each plant species in July. During August and subsequent months, the Pn of *A. adenophora* was suppressed significantly (P < 0.05) with increasing proportions of *H. tuberosus*, whereas the Pn of *H. tuberosus* increased slightly with decreasing proportions of *A. adenophora* in mixed culture (Table 3). Under shaded conditions, the Pn of *H. tuberosus* and *A. adenophora* significantly declined with increasing shade rates, with the inhibition rates of *A. adenophora* higher than those of *H. tuberosus*, showing that shade and plant competition suppressed *A. adenophora* more (Table 4).

**Table 3:** Net photosynthetic rate (Pn) of *Helianthus tuberosus* and *Ageratina adenophora* competition under full sunlight

Variables	Ratios (*H. tuberosus*: *A. adenophora*)				
	4:0	2:1	1:1	1:2	0:4
July					
* H. tuberosus* (µmol CO_2_ m^−2^ s^−1^)	15.59 ± 0.06a	15.65 ± 0.09a	15.57 ± 0.10a	15.69 ± 0.08a	–
* A. adenophora* Pn (µmol CO_2_ m^−2^ s^−1^)	–	9.06 ± 0.08b	9.10 ± 0.06b	9.17 ± 0.03ab	9.27 ± 0.07a
August					
* H. tuberosus* (µmol CO_2_ m^−2^ s^−1^)	18.42 ± 0.09c	18.69 ± 0.07b	18.84 ± 0.06a	18.85 ± 0.03a	–
* A. adenophora* Pn (µmol CO_2_ m^−2^ s^−1^)	–	8.98 ± 0.05d	10.10 ± 0.02c	12.59 ± 0.04b	13.26 ± 0.07a
September					
* H. tuberosus* (µmol CO_2_ m^−2^ s^−1^)	20.11 ± 0.18d	20.80 ± 0.11c	21.49 ± 0.12b	21.83 ± 0.09a	–
* A. adenophora* Pn (µmol CO_2_ m^−2^ s^−1^)	–	9.52 ± 0.12d	11.46 ± 0.13c	13.70 ± 0.13b	15.51 ± 0.17a
October					
* H. tuberosus* (µmol CO_2_ m^−2^ s^−1^)	18.39 ± 0.06c	18.46 ± 0.07bc	18.53 ± 0.07b	18.71 ± 0.05a	–
* A. adenophora* Pn (µmol CO_2_ m^−2^ s^−1^)	–	9.28 ± 0.08d	10.46 ± 0.05c	12.64 ± 0.08b	14.26 ± 0.06a

Data are expressed as mean ± standard deviation. Different letters within the same row signify significant differences at P < 0.05

**Table 4:** Net photosynthetic rate (Pn) of *Helianthus tuberosus* and *Ageratina adenophora* competition under different shade levels

Variables	Different shade rates			
	60%	40%	20%	0%
July				
* H. tuberosus* (µmol CO_2_ m^−2^ s^−1^)				
* H. tuberosus*: *A. adenophora* = 4:0	8.67 ± 0.07d	10.36 ± 0.09c	13.51 ± 0.09b	15.59 ± 0.06a
* H. tuberosus*: *A. adenophora* = 1:1	8.57 ± 0.10d	10.34 ± 0.11c	13.55 ± 0.11b	15.57 ± 0.10a
* A. adenophora* (µmol CO_2_ m^−2^ s^−1^)				
* H. tuberosus*: *A. adenophora* = 1:1	5.05 ± 0.03d	6.05 ± 0.02c	8.80 ± 0.03b	9.10 ± 0.06a
* H. tuberosus*: *A. adenophora* = 0:4	5.13 ± 0.07d	6.21 ± 0.06c	8.83 ± 0.08b	9.27 ± 0.07a
August				
* H. tuberosus* (µmol CO_2_ m^−2^ s^−1^)				
* H. tuberosus*: *A. adenophora* = 4:0	10.81 ± 0.05d	13.11 ± 0.05c	16.65 ± 0.09b	18.42 ± 0.09a
* H. tuberosus*: *A. adenophora* = 1:1	10.67 ± 0.07d	13.63 ± 0.06c	16.88 ± 0.05b	18.84 ± 0.06a
* A. adenophora* (µmol CO_2_ m^−2^ s^−1^)				
* H. tuberosus*: *A. adenophora* = 1:1	6.71 ± 0.03d	7.27 ± 0.08c	9.05 ± 0.01b	10.10 ± 0.02a
* H. tuberosus*: *A. adenophora* = 0:4	7.43 ± 0.04d	9.38 ± 0.04c	11.21 ± 0.04b	13.26 ± 0.07a
September				
* H. tuberosus* (µmol CO_2_ m^−2^ s^−1^)				
* H. tuberosus*: *A. adenophora* = 4:0	11.64 ± 0.09d	13.68 ± 0.08c	17.62 ± 0.12b	20.11 ± 0.18a
* H. tuberosus*: *A. adenophora* = 1:1	12.71 ± 0.05d	15.64 ± 0.07c	19.19 ± 0.13b	21.49 ± 0.12a
* A. adenophora* (µmol CO_2_ m^−2^ s^−1^)				
* H. tuberosus*: *A. adenophora* = 1:1	8.14 ± 0.08d	8.31 ± 0.07c	10.06 ± 0.01b	11.46 ± 0.13a
* H. tuberosus*: *A. adenophora* = 0:4	8.93 ± 0.03d	10.47 ± 0.06c	13.37 ± 0.07b	15.51 ± 0.17a
October				
* H. tuberosus* (µmol CO_2_ m^−2^ s^−1^)				
* H. tuberosus*: *A. adenophora* = 4:0	11.03 ± 0.03d	12.85 ± 0.08c	15.18 ± 0.07b	18.39 ± 0.06a
* H. tuberosus*: *A. adenophora* = 1:1	11.70 ± 0.10d	13.68 ± 0.09c	15.80 ± 0.03b	18.53 ± 0.07a
* A. adenophora* (µmol CO_2_ m^−2^ s^−1^)				
* H. tuberosus*: *A. adenophora* = 1:1	6.52 ± 0.04d	7.55 ± 0.06c	8.88 ± 0.05b	10.46 ± 0.05a
* H. tuberosus*: *A. adenophora* = 0:4	8.24 ± 0.05d	9.64 ± 0.06c	11.99 ± 0.08b	14.26 ± 0.06a

Data are expressed as mean ± standard deviation. Different letters within the same row signify significant differences at P < 0.05

### Competitive interactions

The relative yield (RY) of *H. tuberosus* and *A. adenophora* in different ratios showed that the two plants compete strongly (Tables 5, 6). Under full sunlight, the RY of *H. tuberosus* was significantly higher than 1.0, and the RY of *A. adenophora* was significantly less than 1.0 (P < 0.05) in mixed culture, indicating that the intraspecific competition was higher than interspecific competition for *H. tuberosus*, but the intraspecific competition was less than interspecific competition for *A. adenophora*; the relative yield total (RYT) of *A. adenophora* and *H. tuberosus* was less than 1.0 in mixed culture, indicating that there was competition between the two plants; the competitive balance index (CB) of *H. tuberosus* was greater than zero and the maximum CB index was 1.504 demonstrating a higher competitive ability than *A. adenophora* (Table 5). Under shaded conditions, the RY of *H. tuberosus* markedly declined with increasing shade rates in mixed culture, and the RY of *A. adenophora* was significantly increased with increasing shade rates. However, the CB of *H. tuberosus* was greater than zero, indicating a higher competitive ability than *A. adenophora* even under the highest shade level (60%) (Table 6). Overall, *H. tuberosus* exhibited greater competitive ability than *A. adenophora* under all shade levels (Table 6).

**Table 5:** Relative yield (RY), relative yield total (RYT), and competitive balance (CB) index of *Helianthus tuberosus* and *Ageratina adenophora* under full sunlight

Variables	Ratios (*H. tuberosus*: *A. adenophora*)		
	2:1	1:1	1:2
*H. tuberosus* RYa	1.110 + 0.002b**	1.119 + 0.013b**	1.240 + 0.015a**
*A. adenophora* RYb	0.247 + 0.008c**	0.327 + 0.010b**	0.433 + 0.013a**
RYT	0.678 + 0.003c**	0.723 + 0.009b**	0.837 + 0.009a**
CBa index for *H*. *tuberosus*	1.504 + 0.033a**	1.231 + 0.033b**	1.052 + 0.035c**

Data are expressed as mean ± standard deviation. Different letters within the same row signify significant differences at P < 0.05. The t-test was used to compare each value with 1.0 and 0; * and ** indicate significant differences at 0.05 and 0.01 levels, respectively

**Table 6:** Relative yield (RY), relative yield total (RYT), and competitive balance (CB) index of *Helianthus tuberosus* and *Ageratina adenophora* under different shade levels

Variables	Different shade rates			
	60%	40%	20%	0%
*H. tuberosus* RYa	0.982 + 0.017c**	0.959 + 0.017c**	1.019 + 0.019b**	1.119 + 0.013a**
*A. adenophora* RYb	0.447 + 0.021a**	0.426 + 0.019a**	0.371 + 0.017b**	0.327 + 0.010c**
RYT	0.715 + 0.011a**	0.692 + 0.013b**	0.695 + 0.009b**	0.723 + 0.009a**
CBa index for *H. tuberosus*	0.787 + 0.057c**	0.812 + 0.046c**	1.012 + 0.056b**	1.231 + 0.033a**

Data are expressed as mean ± standard deviation. Different letters within the same row signify significant differences at P < 0.05. The t-test was used to compare each value with 1.0 and 0; * and ** indicate significant differences at 0.05 and 0.01 levels, respectively

## Discussion

The current study demonstrated that compared to *A. adenophora, H. tuberosus* possessed superior attributes in terms of plant height, leaf, biomass, and photosynthesis, and exhibited greater competitive ability than *A. adenophora* under all shade levels when the plants were grown together. Under interspecific competition, morphological characteristics (e.g., leaf shape) and biomass tend to be the most important parameters [25, 26]. Plant species with higher biomass, RY or CB index have stronger competitive ability and are more likely to replace neighboring plants [27, 28]. The underground biomass and aboveground biomass per *H. tuberosus* plant were significantly greater (P < 0.05) than those of *A. adenophora* in all treatments. Our finding that the RY of *A. adenophora* was significantly less than 1.0 in mixed culture under all shade levels, indicated that intraspecific competition was less than interspecific competition for *A. adenophora*. The RY of *H. tuberosus* was significantly less than 1.0 under 40*–*60% and greater than 1.0 under 0*–*20% shade in mixed culture, respectively, showing that intraspecific competition was higher than interspecific competition under low shade rates, but the converse was true under high shade rates. Regardless of shade level, the RYT and CB for *A. adenophora* were significantly less than 1.0, demonstrating that *H. tuberosus* had greater competitive ability than *A. adenophora*. Thus, *H. tuberosus* can provide a promising replacement control candidate for *A. adenophora*.

The initial size of plant individuals and growth stages can affect the competitiveness of a species during interspecific competition [26]. In this study, plant seedlings of *A. adenophora* with 4 leaves and 6*–*7 cm height and slices of *H. tuberosus* tubers with one bud were used to initiate the experiments, which provided *A. adenophora* with an obvious advantage in terms of initial plant height. However, this initial advantage of *A. adenophora* was not sustained during competition with *H. tuberosus* over the season. The lateral expansion rate of *H. tuberosus* seedlings was significantly higher than that of *A. adenophora*. Previous studies observed that plant species with a competitive advantage over *A. adenophora* tended to have characteristics such as rapid growth rate, large leaf area and rapid canopy formation, e.g., *Paspalum wetsfeteini*, *Dolichos lablab*, *Imperata cylindrica*, and *Ipomoea batatas* [28–31]. The high carbohydrate content of *H. tuberosus* tubers, coupled with multiple regenerative strategies featuring vegetative expansion by an extensive rhizome system, and vegetative propagation from tubers, pieces of tubers and rhizomes, can lead to rapid population increases [4]. *Helianthus tuberosus* plants exhibit a rapid increase in plant height, number of leaves and tubers through one life cycle that enable *H. tuberosus* to outcompete most other plant species in arable land [4]. Meanwhile, the plant is also considered a serious weed in some areas because it competes vigorously with other plants in Europe and Canada [4, 15]. After *H. tuberosus* and *A. adenophora* were grown together over the course of a field season, the root biomass, main stem length, leafstalk length, and leaf area of *H. tuberosus* were markedly higher than those of *A. adenophora*, indicating *H. tuberosus* gains the competitive advantage via its strong underground roots and large aboveground individuals.

The leaf is the main site of photosynthesis and leaf area provides a major index to measure growth condition and solar energy utilization efficiency of plants [32]. Greater specific leaf area may contribute to carbon assimilation due to higher leaf area production for a given investment in biomass [33]. *Helianthus tuberosus* and *A. adenophora* are heliophilic species, but may tolerate low sunlight conditions [22, 34]. Our study likewise demonstrated that *H. tuberosus* and *A. adenophora* can survive and grow under high shade rates (as high as 60%). The leafstalk length and leaf area of *H. tuberosus* were markedly greater than those of *A. adenophora* in all treatments. Under full sunlight, the leafstalk length and leaf area of *A. adenophora* progressively declined with increasing proportions of *H. tuberosus*, whereas those of *H. tuberosus* significantly increased with increasing proportions of *A. adenophora* in mixed culture. Similarly, previous studies also showed that the leaf area and Pn of some invasive species were greatly reduced with *I. batatas* competition [31, 35, 36]. The plant growth, biomass, leaf chlorophyll content, photosynthetic rate, transpiration rate, water use efficiency, and stomatal conductance of *A. trifida* were significantly decreased by *H. tuberosus* competition in mixed culture [11, 37]. The Pn of *H. tuberosus* from July to October was higher than that of *A. adenophora*, and the Pn of *A. adenophora* was significantly suppressed with increasing proportions of *H. tuberosus* in mixed culture from August to the end of the growing season. Under various shade levels, the Pn of *H. tuberosus* and *A. adenophora* significantly declined with increasing shade rates, and inhibition rates of *A. adenophora* were higher than those of *H. tuberosus*. Thus, larger leaf area and higher Pn of *H. tuberosus* during the growth period could be responsible for its higher growth rate, branching, and more biomass accumulation in competition with *A. adenophora*.

Competitive plants selected for replacement control should be easy to grow, have high economic value, and possess the ability to form a high canopy density within a short period of time [38]. Because *H. tuberosus* is a multifunctional crop with high ornamental, edible, medicinal, and economic value, it is readily accepted and promoted as an alternative crop [39]. Moreover, this crop is highly resistant to drought, cold temperatures, and saline soil conditions, enabling it to adapt to various climatic and environmental conditions invaded by *A. adenophora* [8, 9]. Our study found that *H. tuberosus* had a higher competitive ability than *A. adenophora* under all shade levels, showing that *H. tuberosus* could be widely used for ecological control in various habitats infected *by A. adenophora*, including shaded orchards and forest edges.

In addition, the competition process is also largely affected by the plant density and planting time [25]. Some studies reported that some replacement plants exhibited a higher CB index at intermediate replacement proportions than at low or high proportions [28, 40]. The plant growth and biomass of *A. trifida* were significantly decreased under different density ratios in mixed culture, and the intraspecific competition of *H. tuberosus* might be more intense than interspecific competition at plant density of 100 plants/m^2^ [37]. Similarly, the current study found that the intraspecific competition was higher than interspecific competition for *H. tuberosus* at a plant density of 20 plants/m^2^ under 0*–*20% shade. *Helianthus tuberosus* grows well in the presence of competitors, and the higher the population density, the sooner the maximum growth rate of each plant was attained [4]. Therefore, in order to optimize the competitive potential of an alternative crop species, a suitable plant density should be selected. Another important recommendation is to plant competitive crops such that they germinate earlier than the weed species of concern [41]. Abundant germplasm resources are available for *H. tuberosus*, and it is generally grown from pieces bearing 1–3 buds, 40*–*70 cm row spacing, 30*–*50 cm plant spacing, and two planting seasons (March–April and September–October). Thus, for replacement control of *A. adenophora*, rational schemes can be designed through row spacing, bud number, variety, and replacement period, in order to enhance the competitive ability of *H. tuberosus*.

## Conclusions

These results showed that as well as being used as a promising alternative crop to outcompete *A. adenophora* under optimal conditions, *H. tuberosus* may also be utilized even in shaded orchards and forest edges invaded by *A. adenophora*. In our experiments, *H. tuberosus* exhibited clear advantages over *A. adenophora* in morphological characteristics, and its competitive ability was significantly higher than that of *A. adenophora* under all shade levels. The *H. tuberosus* crop is a perennial plant with an extensive rhizome system that potentially contributes an even higher competitive advantage with increasing growth years if not harvested annually. Furthermore, as a crop, *H. tuberosus* has many favourable attributes such as nutritional value, ease of propagation, and a variety of medicinal and industrial uses, enabling it to be readily promoted to society as an alternative crop. Further studies of the competitive relationship between *H. tuberosus* and *A. adenophora* would be helpful in providing a stronger basis for utilizing *H. tuberosus* as a competitor, e.g., examining the effects of soil nutrients, enzyme activities and fertility levels.

## Methods

### Study site

The study site was located in Songming County (25° 05′–25° 28′ N; 102°40′–103° 20′ E), Kunming City, Yunnan Province, Southwest China. This area is characterized by a subtropical and temperate monsoon climate. Rainfall averages 1000–1300 mm per year and the annual mean temperature is 14.1 °C. Recently, *A. adenophora* has become widely distributed in orchard lands, wastelands, roadsides, forest edges, and other disturbed ecosystems in Songming County [42].

### Study species

*Helianthus tuberosus* is widely grown as an important food and cash crop in temperate, tropical and subtropical regions in China. This crop mainly reproduces through asexual means and is usually propagated via tubers [6]. Since 2015, various *H. tuberosus* varieties in Yunnan Province have been collected and grown in the greenhouse of the Agricultural Environment and Resource Research Institute, Yunnan Academy of Agricultural Sciences.

*Ageratina adenophora* is one of the most serious invasive species in Yunnan Province, infesting an area of over 300,000 km^2^ [43]. Seeds from local populations of *A. adenophora* were collected in September in 2018, dried at room temperature for two months, and then kept at − 4 °C.

### Experiment design and data collection

The experiments were conducted during the April*–*October 2019 growing season at the Agricultural Environment and Resource Research Institute, Yunnan Academy of Agricultural Sciences, in Xiaojie Town, Songming County, utilizing a de Wit replacement series method [44]. Seeds of *A. adenophora* were propagated in the greenhouse starting on 20 April. On 23 June, the tubers of *H. tuberosus* sown in the greenhouse in 2018 were collected and cut into one-bud pieces. Then, seedlings were planted with consistently the same height (four leaves, 6*–*7 cm) of *A. adenophora* and one-bud pieces with uniform size of *H. tuberosus* were selected. Treatments of 60% (3 layers), 40% (2 layers), 20% (1 layer), and 0% (0 layer, full sunlight, CK) shade rates in this study were created by covering shade houses with different layers of black nylon shade netting. Five ratios of *H. tuberosus* and *A. adenophora* plants (4:0, 3:1, 2:1, 1:1, 1:2, 1:3, 0:4) under full sunlight, and three ratios of *H. tuberosus* and *A. adenophora* plants (4:0, 1:1, 0:4) under three shade levels (60, 40 and 20%) were utilized, respectively, while maintaining a constant overall planting density of 20 plants/m^2^ (0.25 m × 0.20 m space). All plots were arranged in a complete randomized design with 4 replicates utilizing 9 m^2^ plots (3 m × 3 m). All plants were transplanted and distributed evenly within the plot. During the experiment, the plots were weeded and no synthetic fertilizers were used.

From July to October, net photosynthetic rate (Pn) measurements on leaves for *H. tuberosus* and *A. adenophora* were conducted mid-month using a Portable Photosynthesis System (LI-COR LI6400XT), between 8:00 am and 11:30 am, with a 6400-02 or -02B LED source and 1000 μmol m^−2^ s^−1^ photosynthetically active radiation under different sunlight conditions. During sampling, CO_2_ concentration, air temperature and relative humidity (RH) in the chamber were under natural conditions. Measurements were made on a representative leaf randomly chosen on five to six randomly selected individuals of each species.

The experiment was terminated on 22 October 2019, 121 days after the initial transplanting. Twenty plants of each species were selected randomly and harvested from the interior of each plot. Total shoot length, main stem length, branch number, and leafstalk length, were measured with a ruler. Underground and aboveground biomass (fresh weight) were measured using an electronic balance. Leaves were clipped and passed through a leaf-area meter (Li-3000A; Li-Cor Corp.) to determine leaf area index.

### Data analyses

The RY per plant, RYT and CB were calculated from final biomass for each species in each plot. Relative yield per plant of species a or b (i.e., species a and b represented *H. tuberosus* and *A. adenophora* in a mixed culture with species b or a was calculated as RY_a_ = Y_ab_/Y_a_ or RY_b_ = Y_ba_/Y_b_ [44]. Relative yield total was calculated as RYT = (RY_a_ + RY_b_)/2 [45]. Competitive balance index was calculated as CB_a_ = In (RY_a_/RY_b_) [46]. Where Y_ab_ is the yield for species a growing with species b (g/individual), Y_ba_ is the yield for species b growing with species a, Y_a_ is the yield for species a growing in pure culture (g/individual), Y_b_ is the yield for species b growing in pure culture. Values of RY_ab_ measure the average performance of individuals in mixed cultures compared to that of individuals in pure cultures. An RY_ab_ of 1.00 indicates species a and b are both equal in terms of intraspecific competition and interspecific competition. An RY_ab_ greater than 1.00 means intraspecific competition of species a and b is higher than interspecific competition, and an RY_ab_ of less than 1.00 implies intraspecific competition of species a and b is less than interspecific competition. Relative yield total is the weighted sum of relative yields for the mixed culture components. An RYT of 1.00 means that both species are competing for the same resources, and one is potentially capable of excluding the other; an RYT of greater than 1.00 means that the two species exploit different resources and therefore do not compete (e.g., due to different root depths); finally, an RYT of less than 1.00 implies that the two species are mutually antagonistic, with both having a detrimental effect on the other [45]. Values of CB_a_ greater than 0 indicate that species a is more competitive than species b [46].

All morphological variables (total shoot length, branch number, leaf area, leafstalk length, and biomass), as well as photosynthetic rate (Pn) of *H. tuberosus* and *A. adenophora* plants were analyzed by analysis of variance (two-way ANOVA) using IBM SPSS 23.0 software (Armonk, New York, USA). If significant differences were detected with the ANOVA, Duncan’s multiple range tests were used to detect differences among treatments at a 5% level of significance. Relative yield and RYT from each mixed culture were compared to the value of 1.00 using one sample t-tests (P = 0.05), and values of RYT were tested for deviation from 1.0 and values of CB for deviation from 0 using a paired t-test.

## Data Availability

The data set supporting the results of this article is available in the Dryad Digital Repository https://doi.org/10.5061/dryad.s4mw6m96c.
